# Using Quality Measurement to Support Healthy Aging: The Adult Immunization Status Composite Measure

**DOI:** 10.1093/geroni/igaa057.707

**Published:** 2020-12-16

**Authors:** Courtney Barbera

**Affiliations:** Discern Health, Baltimore, Maryland, United States

## Abstract

Vaccine-preventable diseases remain a significant public health threat, especially for older adults and those with compromised immunity. Efforts to drive vaccine uptake can include quality measures, which help providers, health care organizations, payers, and others to benchmark and track immunization activities. To help address low immunization rates in adults, the National Committee for Quality Assurance (NCQA) released the Adult Immunization Status (AIS) composite measure, which calculates a single rate for health plan performance on influenza, Td/Tdap, shingles, and pneumococcal immunizations. Discern Health and the American Medical Group Association (AMGA) performed a mixed-methods study using de-identified patient data from three healthcare organizations to analyze feasibility of the AIS measure in quality programs at the medical group-level. The team also team conducted qualitative interviews with the organizations to understand best practices for improving immunization rates in adults. The study showed that the AIS measure is feasible for use at the medical-group level. It also revealed that adults 50 years of age and older were less likely to receive all age-appropriate vaccinations than those younger than 50. These findings indicate that there is a significant gap in rates of immunizations among older adults and point to potential solutions that can be supported through use of the AIS measure.

